# Prevalence of venous extension in malignant adrenal neoplasia: beyond primary tumors and identification of a novel imaging sign

**DOI:** 10.1007/s00330-026-12382-1

**Published:** 2026-02-28

**Authors:** Lais P. D. M. Melges, Cecília V. S. Torres, Fernando Chahud, Jorge Elias, Leandro M. Colli, Carlos A. F. Molina, Margaret Castro, Valdair F. Muglia

**Affiliations:** 1 Department of Medical Images, Oncology and Hematology, Ribeirao Preto School of Medicine—University of Sao Paulo (USP), São Paulo, Brazil; 2 Department of Pathology, Ribeirao Preto School of Medicine—University of Sao Paulo (USP), São Paulo, Brazil; 3 Department of Surgery — Urology Division, Ribeirao Preto School of Medicine—University of Sao Paulo (USP), São Paulo, Brazil; 4 Department of Internal Medicine — Endocrinology Division, Ribeirao Preto School of Medicine—University of Sao Paulo (USP), São Paulo, Brazil

**Keywords:** Adrenal cancer, Adrenocortical carcinoma, Metastases, Magnetic Resonance Imaging, Computed Tomography

## Abstract

**Objective:**

To assess the prevalence of adrenal vein involvement in primary and metastatic adrenal lesions and to determine if morphological changes in tumor shape precede venous extension.

**Materials and methods:**

This retrospective, single-center observational study evaluated 102 patients: 28 adrenal cortical carcinoma (ACC) patients, and 74 non-ACC cancer patients that presented adrenal metastasis (82 metastatic adrenal lesions). Two readers reviewed cross-sectional imaging to assess tumor size, laterality, venous invasion, and the presence of the “edge sign.” Surgical and histopathological confirmation was the reference standard for ACCs, while for metastases, sequential imaging or PET-CT results showing hypermetabolism were used in 70.7% of cases and histopathology in 29.3% of cases.

**Results:**

Of the 28 ACC patients, 82.1% were female, with balanced laterality. Metastases primarily originated from the lung (24.4%), colorectal (13.4%), and breast (12.2%) cancers and had a left-sided dominance (61.7%). Venous extension was present in 14.6% of metastases and 21.4% of ACCs, a non-significant difference (*p* = 0.40). The “edge sign” was more frequently observed in metastatic lesions than in ACCs, 26.8 × 17.8%, although this difference has not reached statistical significance (*p* = 0.34). In multivariate analysis, both mean size and the “edge sign” were independent predictors of adrenal and renal vein extension. Interobserver agreement was almost perfect for venous extension (κ = 0.9256) and substantial for the edge sign (κ = 0.7844).

**Conclusion:**

Venous extension was less prevalent in metastatic adrenal lesions compared to ACCs. The edge sign may precede venous extension, especially in metastatic cases, indicating the nature of the lesion. These findings potentially may alter disease management, expediting the decision for surgery; however, prospective multicenter studies are needed to confirm their clinical impact.

**Key Points:**

***Question*** *What is the prevalence of venous extension in malignant adrenal lesions—whether primary or secondary—and how can early involvement be recognized on imaging?*

***Finding**** Adrenal vein involvement occurred similarly in ACCs (21.4%) and metastases (14.6%). Early extension may be preceded by the adrenal edge sign in 25% of cases*.

***Clinical relevance**** Adrenal vein involvement occurs in both primary and metastatic adrenal lesions, with a tendency to be more prevalent in adrenocortical carcinomas. The ‘edge sign’ may precede venous extension in malignant lesions, aiding both diagnosis and therapeutic planning*.

**Graphical Abstract:**

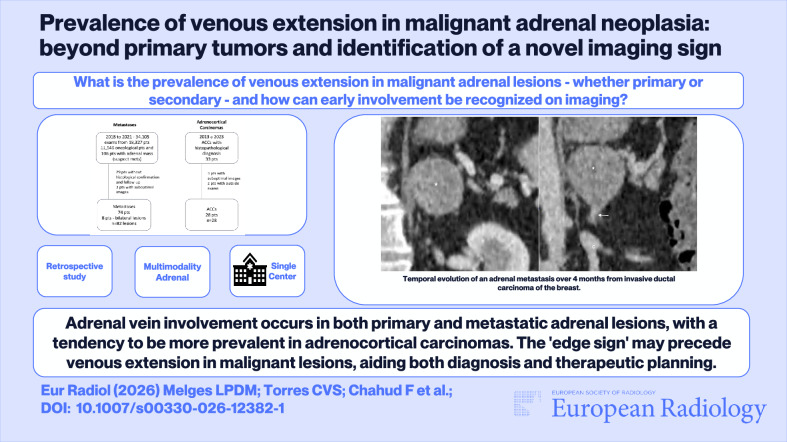

## Introduction

Adrenal cortex carcinoma (ACC) is a rare neoplasm that encompasses a heterogeneous group of tumors. Due to its rarity, establishing consensual guidelines in the literature has been difficult [1]. Recent data from the United States places its incidence at around 0.8 per million inhabitants [2], while European data suggest a slightly higher incidence [3]. Research in Brazil has identified the p.R337H mutation in the TP53 gene as significantly associated with adrenal tumors, leading to an incidence 8–15 times higher than the global average. This mutation is notably prevalent in Southern Brazil and has implications for familial cancer syndromes, such as the Li-Fraumeni syndrome and its variants [4].

The diagnosis of adrenal cortex malignant neoplasia is based on a combination of signs and symptoms, laboratory changes, and imaging tests, being classified in functioning or non-functioning tumors [5]. Imaging findings are always fundamental, but they are especially crucial in non-functioning adrenal tumors where staging is vital for both surgical planning and prognostic assessment [6, 7]. The rarity of these lesions has long hindered consensus in international guidelines. However, the European Network for the Study of Adrenal Tumors (ENSAT) recently proposed standardized criteria for both diagnosis and staging [8].

A key aspect of adrenal tumor staging is identifying the tumor’s extension into surrounding structures such as the adrenal vein, renal vein, and, more rarely, the inferior vena cava (IVC) or even the right atrium [9]. Although adrenocortical carcinoma (ACC) is already rare, venous extension is an even less common occurrence. Studies estimate that venous involvement occurs in 9–19% [10] of all cases. While venous involvement in ACC is well-documented, reports of such involvement in metastases are infrequent in the literature [11]. Historically, tumor extension into the adrenal and renal veins has been associated primarily with primary adrenal malignancies. However, more recent studies suggest that venous involvement can also be seen in metastatic adrenal lesions and, more rarely, in conditions like pheochromocytoma or neuroblastoma [12, 13]. In a retrospective analysis of 246 surgically treated adrenal masses, Osman et al [13] reported that 2.9% of patients showed extension into the adrenal or renal veins. This included cases of ACC but also instances of neuroblastoma and pheochromocytoma.

Both from a therapeutic (surgical) planning standpoint and in terms of prognosis for both primary and secondary lesions, defining adrenal vein involvement is critical. For example, the resection of isolated adrenal metastases positively impacts survival in patients with colorectal neoplasia [14]. Therefore, it is extremely important that imaging approaches for metastases also detail the presence and extent of venous involvement similar to how venous extension is assessed in other organs—such as in renal cell carcinoma, where it often begins with partial filling defects at the level of the renal hilum [15].

Noticing that adrenal lesions often exhibited a characteristic morphological change on their inferior surface before reaching the adrenal vein, our group hypothesized that this could be a sign of early invasion and could be used to identify patients at risk for adrenal dissemination. Although there is no specific mention of this phenomenon in the literature—neither for the adrenal glands nor for other organs—some authors [15, 16] have shown that pancreatic neuroendocrine tumors may extend into the portal, splenic, or superior mesenteric vein (SMV), altering the vessel’s contour, particularly when involving the SMV.

Accordingly, our goals here were twofold. First, given the limited research on how often adrenal vein involvement occurs in both primary and metastatic adrenal lesions, to determine its frequency. And second, to assess whether morphological changes in tumor shape precede venous extension in these cases.

## Patients and methods

### Patient selection

This retrospective observational study was conducted at a single tertiary care, a referral center for endocrinological diseases and urinary oncology. The study was approved by the Ethics and Research Committee of the Clinical Hospital at Ribeirao Preto Medical School under number CAAE 78221024.2.0000.5440, with a waiver from the Informed Consent Form.

We searched for patients with primary malignant neoplasms confirmed by histopathological findings and who were treated between 2013 and 2023. We also included patients with metastasis or lesions suspected of metastasis based on imaging studies between 2018 and 2021. These patients were followed up for undergoing oncological disease and/or had PET/CT scans showing a new adrenal lesion, and had further histopathological confirmation. The time search was restricted to cases until 2021 to allow a sufficient follow-up period to confirm the nature of the lesions. In this way, the exclusion criteria were either loss of follow-up in suspected metastatic cases, or inadequate exams for patient evaluation.

**Fig. 1:**
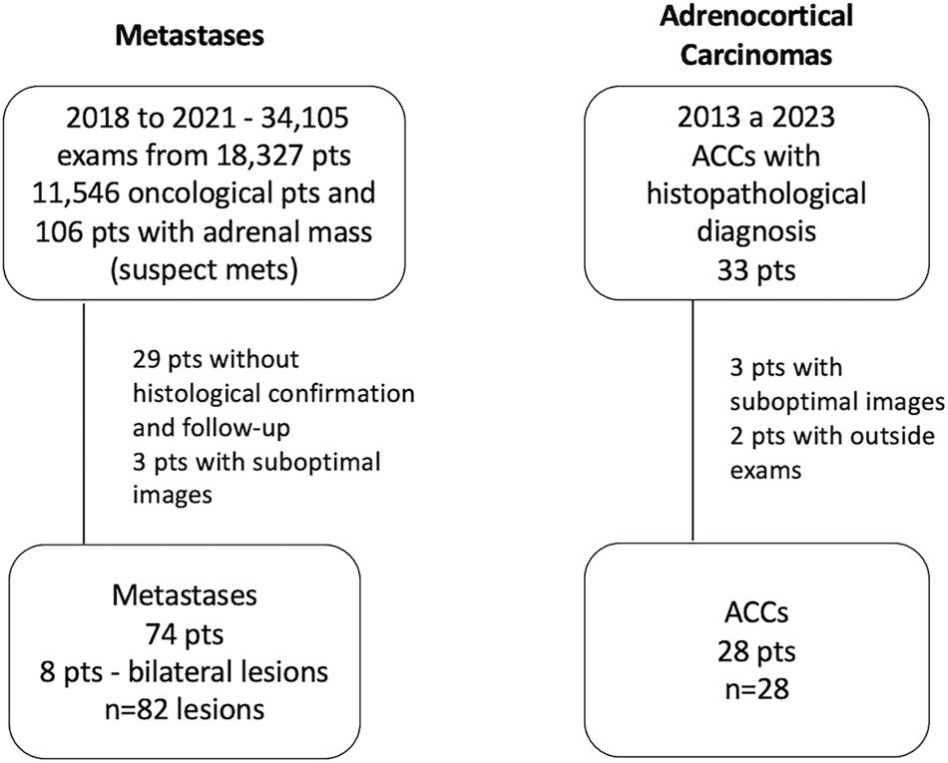
Flow chart showing the selection of patients, exclusion criteria, and final number of patients and cases. ACC, adrenal cortical carcinoma; pts, patients

Accordingly, 139 patients were identified, 33 with ACC and 106 with lesions suspected of metastases. Among the 33 patients with ACC, 3 were excluded due to inadequate exams, and 2 had no imaging exams from our institution, leaving a total of 28 patients. Among the 106 patients with suspected metastasis, 29 had only one imaging exam, without follow-up or histological confirmation, and 3 had exams with significant artifacts, preventing analysis, leaving a total of 74 patients. Eight of these patients had bilateral lesions, resulting in a total sample of 82 lesions suspected of metastases (Fig. 1).

### Imaging protocols

Four CT scanners were used in the study: three from Canon, one Aquilion Prime, and two Aquilion One, with 80 detectors and 160 sections, and one Brilliance scanner from Philips with 16 detectors. CT images were acquired with 1.0 mm thin sections and reconstructed with 2.0 mm thickness. The protocols varied based on the clinical indication, including an adrenal protocol with a 15-min delayed phase or a staging and/or follow-up protocol for oncologic patients, which involved three-phase acquisition.

For the MRI exams, we used two 1.5-Tesla scanners: one Achieva and one Ingenia, both from Philips. The MRI protocol included axial T1 images in-phase and out-of-phase, axial, coronal, and sagittal T2 images, and diffusion-weighted images with b-values ranging from 50 to 1000 mm²/s. The post-contrast images were acquired at 30, 60, and 120 s.

### Image analysis

The images of the included patients were reviewed by two independent Abdominal radiologists - one with five years and the other with four years of experience in Abdominal imaging. The radiologists were blinded to the specific diagnosis of the lesions but were aware of the study’s objective. They analyzed the images at a dedicated workstation using HOROS DICOM Viewer version 3.3.6. The following data were collected: (a) laterality; (b) dimensions; (c) signal attenuation/intensity coefficient; (d) density or signal pattern (homogeneous, slightly heterogeneous, or predominantly heterogeneous); (e) extension to the renal and adrenal veins; and (f) changes in the shape of the lesion on its lower surface, referred to as the “edge sign” when seen on coronal and or sagittal images.

**Fig. 2:**
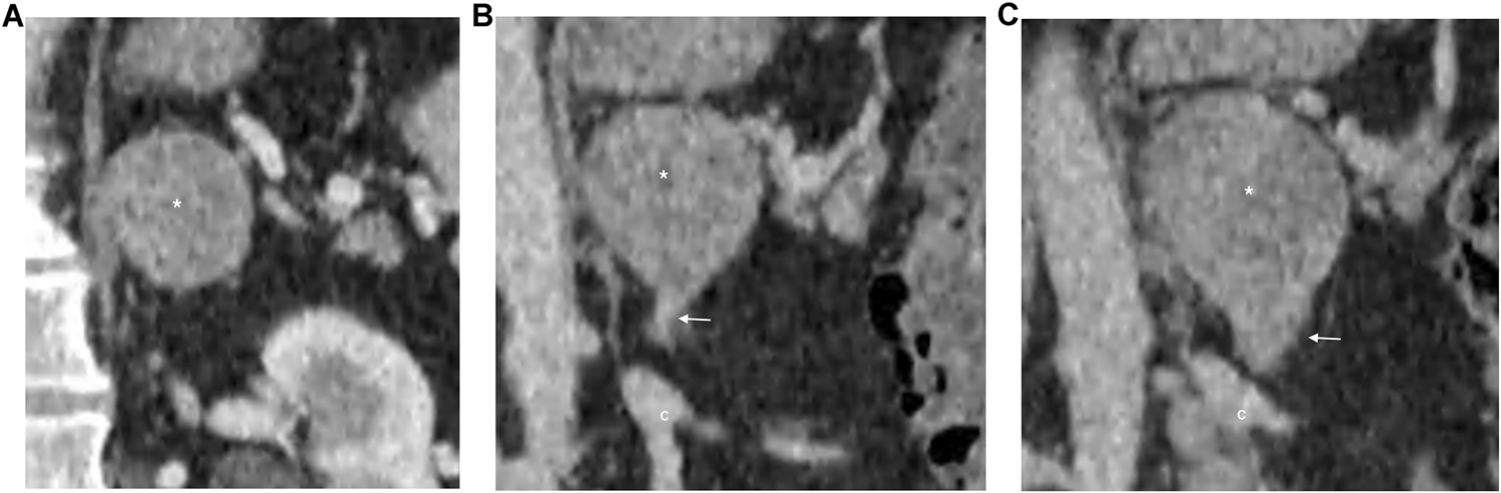
A forty-eight-year-old female had an Invasive Ductal Carcinoma of left Breast resected 6 years ago. All images after intravenous contrast media, in coronal reformation. **A** A heterogeneous mass (*) is seen on follow-up CT in June. The lesion (*) has a round format. **B** Abdominal CT, Oct of the same year. The lesion has grown but has changed its format, with an indentation in its inferior aspect. The left adrenal vein (arrow) is seen slightly enlarged, with enhancement similar to other vessels. The left renal vein is seen at a short distance, at the point of the confluence (**C**) with the gonadal vein. **C** Mar the following year. The lesion (*) is in continuity with the left adrenal vein (arrow), which is markedly enlarged now, with attenuation identical to the tumor and growing towards the renal vein, at the level of the confluence of the gonadal with renal vein (**C**)

The criteria for venous involvement included enlargement of the adrenal and/or renal vein, with internal content displaying attenuation or signal intensity similar to that of the adrenal lesion, along with enhancement following intravenous contrast administration [17, 18]. The “edge sign” was defined as a sharp indentation on the inferior aspect of the adrenal lesion, visible on either the sagittal or coronal plane (Fig. 2). For patients with metastases, follow-up exams were reviewed to determine if the adrenal or renal veins became involved after a prior exam had shown the presence of the “edge sign.”

Discrepancies in both cases were resolved through consensus, involving a senior abdominal radiologist with over 20 years of experience in the field.

### Clinical data

Another observer, a radiologist with 5 years of experience, who was not involved in the image analysis, retrieved the patients’ clinical data (gender and age) and anatomopathological information through the electronic medical record system.

### Reference standard

The reference standard for ACC was histopathological confirmation. For metastases, when histopathological confirmation was not possible, the appearance of new adrenal lesions during sequential evaluations or PET-CT scans showing hypermetabolism of these lesions in patients previously diagnosed as primary tumors was used as an alternative reference. To assess the venous involvement in patients with anatomopathological confirmation, the anatomopathological findings were reviewed by an experienced pathologist with over 15 years of expertise in urinary tract diseases. In addition to histopathological analysis, a review of surgical specimens was conducted to determine the presence or absence of adrenal vein extension (AVE) during surgery. For the cases of metastases without histopathological diagnosis through an adrenalectomy, we use consecutive exams showing progression of adrenal venous extension to the renal vein.

### Statistical analysis

For statistical analysis, Stata software, version 15, was used. Demographic data were described using the mean, median, and standard deviation. Categorical variables were described by proportions and their respective confidence intervals. The parametric ANOVA test was used to compare numerical variables, while the Kruskal-Wallis test was applied for non-Gaussian distributions. Interobserver agreement was calculated using Cohen’s method, with the following interpretation for kappa values: between 0.0 and 0.20, weak agreement; 0.21 and 0.40, fair agreement; 0.41 and 0.60, moderate agreement; 0.61 and 0.80, substantial agreement; and 0.81 and 1.0, almost perfect agreement [19]. In this study, a *p*-value of less than 0.05 was set as the threshold for statistical significance.

## Results

The mean age of patients with metastases was 64.1 ± 9.7 years, while for ACC patients it was 24.7 ± 25.2 years. ACC had a significantly higher prevalence in females, accounting for 82.1% of the cases, in contrast to patients with metastases, who showed no clear sex predominance. ACCs exhibited a more balanced laterality, with 46.4% occurring on the right and 53.6% on the left, whereas in patients with metastasis, lesions were more frequent on the left side (61.7%). All cases of ACC were confirmed by histopathological examination. Regarding metastases, 58 out of 82 lesions (70.7%) were confirmed through follow-up exams, while 24 (29.3%) were confirmed by histopathological findings.

The mean longest axis for patients with metastasis was 4.1 ± 2.6 cm according to reader 1 and 5.3 ± 2.5 cm according to reader 2. For patients with ACCs, the average size was 7.3 ± 4.2 cm for reader 1 and 8.3 ± 4.4 cm for reader 2, as detailed in Table 1.

**Table 1:** Clinical, demographic, and pathological data of both groups

		METs	ACCs	*p*-value
Age		64.01 ± 9.7	24.7 ± 25.2	< 0.0001
Sex	F	39 (52.7%)	23 (82.1%)	0.006
	M	35 (47.3%)	5 (17.9%)	
Side	Right	31 (38.3%)	12 (42.8%)	0.67
	Left	51 (61.7%)	16 (51.2%)	
Size (mm)	Reader 1	4.1 ± 2.6	7.3 ± 4.2	< 0.0001
	Reader 2	5.3 ± 2.5	8.3 ± 4.4	< 0.0001
Standard of reference	F/UP/exams	58 (70.7%)	0	0.001
	Surgery + AP	24 (29.3%)	28 (100%)	
Staging		IV	ENSAT I (*n* = 10–35.7%) II (*n* = 7–25.0%) III (*n* = 9–32.2%) IV (*n* = 2–07.1%)	N/A

*ACCs* adrenal cortical carcinomas, *AP* anatomopathological diagnosis, *METs* metastases, *F/UP* follow up, *ENSAT* European Network for the Study of Adrenal Tumors

The most prevalent primary site for metastasis in this study was the lung (24.4%), followed by colorectal neoplasms (13.4%) and breast cancer (12.2%), as shown in Table 2. All patients with adrenal metastases presented with Clinical Stage IV. Some had multiple sites of involvement, while others had isolated adrenal lesions. The ENSAT staging for ACC is shown in Table 1. The most frequent was stage I (*n* = 10) with 35.7%, followed by stage III, 9 patients (32.2%). Only 2 patients (7.1%) were stage IV.

**Table 2:** Data about metastasis, venous extension, and edge sign

	Total *n* = 82	Venous extension *n* = 12	Edge sign *n* = 22
Lung	20 (24.4)	3 (25.0)	8 (36.4)
Colorectal	11 (13.4)	4 (33.4)	5 (22.8)
Breast	10 (12.2)	3 (25.0)	5 (22.8)
Stomach	9 (11.0)		1 (4.5)
Renal cell carcinoma	7 (8.6)		
Melanoma	5 (6.1)	1 (8.3)	1 (4.5)
Endometrium	4 (4.9)	1 (8.3)	1 (4.5)
Urothelial	4 (4.9)		1 (4.5)
Esophagus	2 (2.4)		
Liver	2 (2.4)		
Prostate	2 (2.4)		
Uterine (cervix)	2 (2.4)		
Oropharinx, pancreas, parotid, kaposi	1 (1.2)		

### Venous extension

Venous extension to the adrenal vein was observed in 14.6% (12 lesions) of patients with metastases and 21.4% (6 lesions) of ACC patients, a non-significant difference (*p* = 0.40). Metastases originating from colorectal cancer had the highest rate of adrenal vein involvement (33.4%), followed by lung (25%) and breast (25%) cancers. We carried out a sensitivity analysis considering venous extension in the subgroup of histologically confirmed metastases. The prevalence in this subgroup—16.6%—was not significantly different from that observed in the overall metastasis group (*p* = 0.81).

All 28 patients with ACC included in the study underwent surgery and histopathological analysis, which confirmed the venous status in all cases. In contrast, among the patients with metastases, histopathological confirmation was available for only 19 cases; 10 of these patients underwent adrenalectomy, and just one showed imaging signs of venous extension. In these 10 patients, the imaging-pathology correlation was flawless, with no false positives or false negatives. Of the 74 patients with metastatic lesions, only 9 had isolated adrenal metastases, while the remaining 65 had adrenal involvement along with other sites.

**Fig. 3:**
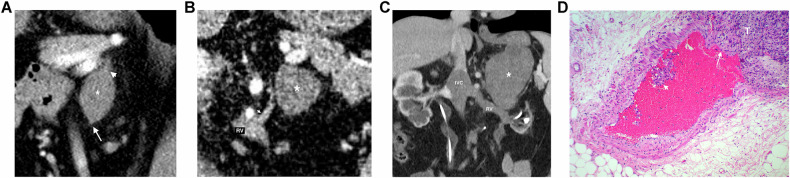
A 61-year-old male patient on follow-up for a resected rectal cancer appears with a mass in the left adrenal gland (asterisk in images **A**–**C**), confirmed histologically as a metastatic lesion. **A**, **B** CT from Aug. **A** Axial image showing a predominantly exophytic lesion arising from the adrenal (arrowhead) seen in the sagittal plane, where it’s possible to see an indentation on the inferior aspect, the Edge sign (arrow). **B** CT coronal image, it is possible to see the lesion extending close to the exit of the adrenal vein (arrow), which is dilated, but with a similar enhancement pattern (arrowhead) as the renal vein (RV). **C** CT scan of Feb of the following year. In the coronal plane, a significant increase in the size of the lesion is seen. The adrenal vein is more dilated, now with hypodense content and enhancement due to extensive tumor thrombosis that extends throughout the RV to the IVC. **D** Histological section (hematoxylin & eosin—100×) showing the tumor (T) invading the wall (arrow) of a tributary of the adrenal vein, with the tumor already within the lumen (arrowhead)

The “edge sign” (Fig. 3) was observed in 22 out of the 82 metastases (26.8%) and in 5 out of the 28 ACC cases (17.8%), a non-significant difference (*p* = 0.34), as shown in Table 2. Among patients with metastases, the edge sign was most frequently observed in lung (36.4%), colorectal (22.8%), and breast (22.8%) neoplasms, as shown in Table 2. Among the 22 patients with metastases and an edge sign, 12 (54.5%) developed confirmed venous extension during follow-up.

**Fig. 4:**
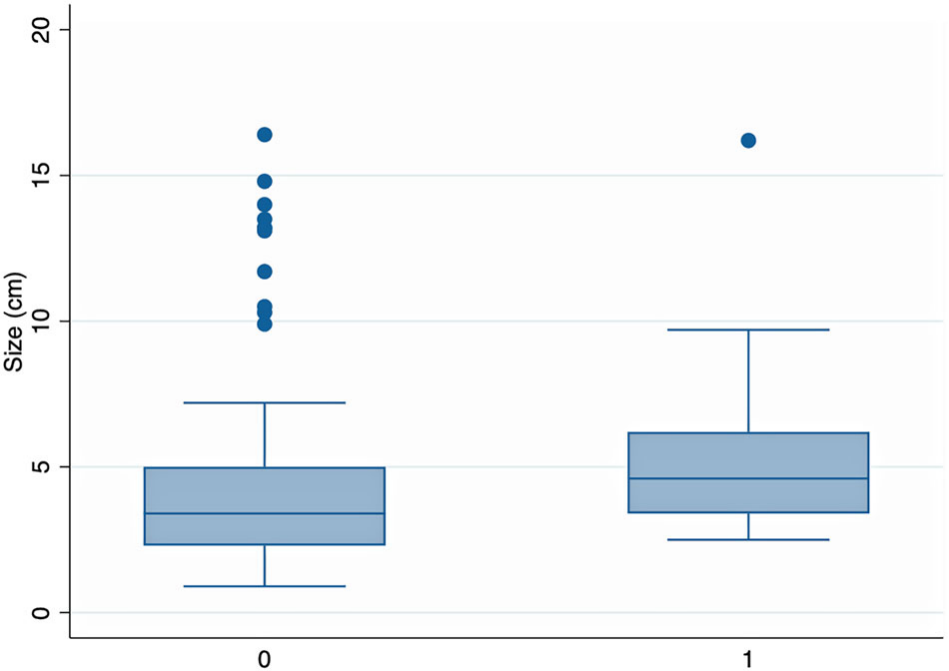
Scatter plot showing the distribution of lesion size (in centimeters) and the presence of the edge sign. 0 = absence; 1 = presence of Edge Sign. *Lesion size—the mean values of both readers

At regression analysis, the mean size of lesions (Fig. 4) and the “edge sign” were independent predictors of AVE and renal vein extension (RVE), at uni and multivariate analysis. However, the edge sign was a stronger predictor for both AVE and RVE (Table 3).

**Table 3:** Regression analysis showing independent predictors of AVE and RVE, detected by imaging

	AVE	*p*-value	RVE		*p*-value
Univariate analysis					
Sex	0.03 ± 0.51	0.94	−0.89 ± 0.67		0.81
Mean size*	0.18 ± 0.06	0.005	0.17 ± 0.07		0.01
Edge sign	3.86 ± 0.70	0.0001	2.81 ± 0.64		0.0001
Multivariate analysis					
Sex	−0.89 ± 0.67	0.18	−1.35 ± 0.80		0.09
Mean size*	0.17 ± 0.07	0.01	0.21 ± 0.09		0.02
Edge sign	2.81 ± 0.84	0.0001	2.87 ± 0.69		0.0001

* Average values between two readers were used for regression analysis

*AVE* adrenal vein extension *RVE* renal vein extension

### Interobserver agreement

The interobserver agreement for venous extension to the adrenal vein had a Kappa coefficient of 0.9256 (*p* < 0.001), indicating almost perfect agreement. For the “edge sign,” the interobserver agreement had a Kappa coefficient of 0.7844 (*p* = 0.001), representing substantial agreement (Table 4).

**Table 4:** Interobserver agreement for venous extension and the edge sign

	Agreement	Expected agreement	Kappa	*p*-value
Venous extension	98.6%	81.3%	0.9256 ± 0.08	0.0001
Edge sign	93.1%	67.8%	0.7844 ± 0.08	0.001

## Discussion

The scarce data on the prevalence of AVE in primary and metastatic adrenal lesions do not contribute to the widespread use of imaging studies for differentiating between these two types of lesions. To address this, we evaluated adrenal vein involvement in patients with adrenal lesions from metastases or ACC. Our data, based on sequential histopathological analysis or imaging follow-up, showed similar rates of vein involvement between the two groups: 14.6% in metastases and 21.4% in ACC. Notably, metastases from colorectal cancer had the highest rate of adrenal vein invasion (33.4%), followed by lung and breast cancers.

In our cohort, the prevalence of venous extension in ACCs was 21.4%, slightly above the range of 9–19% described by Ng and Libertino [10]. One possible explanation could be a selection bias toward more aggressive cases, as our institution is a referral center for pediatric and adult adrenal tumors.

In our series, we found venous extension in 14.6% of patients with metastases. However, this rate may be underestimated, as most metastases were confirmed through imaging follow-up. Even in cases with histological confirmation, the diagnosis was based on biopsy rather than adrenalectomy, which may have missed microscopic or early venous extension.

Moreover, there is limited data in the literature regarding the prevalence of venous extension detected through imaging [20], or histopathological data from surgical specimens and/or autopsies [21]. In a recent review, Ferriero et al [22] assessed the impact of primary tumor histology on survival outcomes following minimally invasive adrenalectomy for solitary metachronous metastasis. Of the 235 adrenalectomies with pathology-confirmed metastasis, 60 did not mention venous involvement. Similarly, Banks et al [23], in their systematic review of the literature on adrenal biopsy performance in diagnosing adrenal malignancies, also did not report any invasion of the adrenal vein by metastatic lesions.

The “edge sign” was identified in a higher percentage of metastatic lesions compared to ACCs (26.8% vs 17.8%). One possible explanation is that metastatic lesions, due to long-term systemic treatment, often undergo extended follow-up. These patients typically have imaging at various time points, allowing analysis of metastatic lesions from the time they are confined to the adrenal gland to the onset of venous extension. This enables the detection of when the edge sign appears. In contrast, ACC treatment is primarily surgical, limiting the opportunity for ongoing monitoring, with imaging capturing only the moment the condition is diagnosed. Another noteworthy consideration is that the contour alteration observed on the inferior aspect of the lesions may plausibly be attributed to the anatomical origin of the adrenal vein in the gland’s most caudal portion and its subsequent course toward the RV.

An interesting observation from cross-sectional images is the higher number of cases with the edge sign compared to cases with venous involvement. This suggests that the morphological change may precede venous involvement. However, we cannot entirely rule out the possibility of false positives or false negatives. For the 10 patients with metastasis who underwent adrenalectomy, the imaging-pathology correlation was perfect, with 9 true negative cases and one true positive case for adrenal venous involvement.

The most common primary source of adrenal metastases observed in our study was the lung, in agreement with previous studies [24]. Indeed, both non-small and small cell lung cancer frequently metastasize to the adrenal glands [24, 25]. Other common sources of adrenal metastases observed in our study were colorectal and breast neoplasia. Thus, our study is consistent with previous studies, in which the most frequent primary lesions were in the lung, followed by breast and colorectal neoplasms [26]. In our study, renal cell carcinoma accounted for 9 of the 82 metastatic lesions. In some studies, this primary location is more prevalent than breast and colorectal neoplasms [24, 27].

The reproducibility of the analysis between the two readers in the assessment of lesion size and for venous extension and the edge sign was very high, which is encouraging for its application in clinical practice, if externally validated. It is worth noting that ACC lesions were larger than metastatic lesions. This is likely because a significant number of ACC cases are asymptomatic non-functional tumors and, when diagnosed, the tumors have already reached a considerable size [28], limiting the potential for evaluation of the edge sign, since the large size of the lesion may obscure minor deformities on the lower surface.

There are some limitations to our study. First, the retrospective nature of the study introduces an inherent selection bias. To mitigate this, we applied strict inclusion and exclusion criteria. Additionally, as this is a single-center study, the generalizability of the results may be limited. However, given that this is a hypothesis-generating study, the use of a single center is reasonable for future validation studies. Some of the results are based on subjective analysis, but the high level of agreement between the two observers supports the reproducibility of the criteria used to characterize venous invasion and the deformity of the lower surface of lesions involving the adrenal vein (the edge sign). The relatively low number of ACC cases can be attributed to the extremely low incidence of this neoplasm. We also included pediatric ACCs, which differ in some important histological and clinical aspects from adult ACCs [29]. However, we focused on venous extension and the edge sign, which may be common to both pediatric and adult cases. Given that our sample size is small and the ideal number is practically impossible to achieve due to the extremely low prevalence of primary adrenal tumors, we must consider that the lack of a statistically significant difference in venous extension between ACC and metastases may represent a Type II error rather than true equivalence. Also, we did not obtain histological confirmation for the majority of the metastatic lesions included in the study. Nevertheless, two points should be emphasized: first, histological confirmation of secondary lesions is not always obtained in cancer patients, as it is typically reserved for cases where diagnostic uncertainty exists and confirmation is essential for determining the therapeutic approach [30, 31]. Second, in a significant portion of the metastases extending to the adrenal vein, we did obtain histological confirmation, which strengthens the validity of our data. Last, we used four different CT scanners, and the exams were performed using various protocols, including differences in acquisition timing and peak tube voltage, which may affect the attenuation of both organs and thrombus [32].

In conclusion, our study showed that assessing tumor size, venous involvement, and the edge sign was very reproducible in a clinical scenario. The prevalence of venous extension in metastatic adrenal lesions was lower than that observed in ACCs, suggesting this sign could be tumor-specific. The edge sign may precede venous extension, particularly in metastatic cases. Given the scarcity of such data in the literature, these findings are valuable, although prospective, multicenter studies are needed to validate our findings in metastatic lesions and ACCs and evaluate their potential impact on patient management.

## Abbreviations

ACC: Adrenocortical carcinoma
AVE: Adrenal vein extension
IVC: Inferior vena cava
RVE: Renal vein extension
SMV: Superior mesenteric vein
